# Cell polarity defines three distinct domains in pancreatic β-cells

**DOI:** 10.1242/jcs.185116

**Published:** 2017-01-01

**Authors:** Wan J. Gan, Michael Zavortink, Christine Ludick, Rachel Templin, Robyn Webb, Richard Webb, Wei Ma, Philip Poronnik, Robert G. Parton, Herbert Y. Gaisano, Annette M. Shewan, Peter Thorn

**Affiliations:** 1 School of Biomedical Sciences, University of Queensland, St Lucia, Queensland 4072, Australia; 2 Charles Perkins Centre, John Hopkins Drive, University of Sydney, Camperdown, New South Wales, 2050, Australia; 3 Centre for Microscopy and Microanalysis, University of Queensland, St Lucia, Queensland 4072, Australia; 4 Department of Physiology, School of Medical Sciences, The University of Sydney, Camperdown, New South Wales, 2006, Australia; 5 Institute for Molecular Bioscience, University of Queensland, St Lucia, Queensland 4072, Australia; 6 Department of Medicine, University of Toronto, Toronto, Ontario, M5S1A8, Canada; 7 School of Chemistry and Molecular Biosciences, University of Queensland, St Lucia, Queensland 4072, Australia

**Keywords:** Insulin, Polarity, Islet, Diabetes, β-Cell

## Abstract

The structural organisation of pancreatic β-cells in the islets of Langerhans is relatively unknown. Here, using three-dimensional (3D) two-photon, 3D confocal and 3D block-face serial electron microscopy, we demonstrate a consistent *in situ* polarisation of β-cells and define three distinct cell surface domains. An apical domain located at the vascular apogee of β-cells, defined by the location of PAR-3 (also known as PARD3) and ZO-1 (also known as TJP1), delineates an extracellular space into which adjacent β-cells project their primary cilia. A separate lateral domain, is enriched in scribble and Dlg, and colocalises with E-cadherin and GLUT2 (also known as SLC2A2). Finally, a distinct basal domain, where the β-cells contact the islet vasculature, is enriched in synaptic scaffold proteins such as liprin. This 3D analysis of β-cells within intact islets, and the definition of distinct domains, provides new insights into understanding β-cell structure and function.

## Abstract (precis)

**Summary:** 3D imaging methods identify three structural and functional domains within β-cells in islets: apical, lateral and basal.

## INTRODUCTION

Cell polarity is established in response to external cues, and drives cell orientation and regional specialisations that are essential for cell function (Roignot et al., 2013; Yu et al., 2005). Apical-basal polarity determinants define intracellular domains and create membrane segregation (Bryant et al., 2010). These domains are then the target for the location and trafficking of specific proteins (Cao et al., 2012; Mellman and Nelson, 2008) that in turn are crucial for function. Perhaps the best known class of cells that are polarised are epithelial cells. For example, in pancreatic acinar cells, apical location of the exocytic machinery (Gaisano et al., 1996) and Ca^2+^ release apparatus (Thorn et al., 1993) are essential for the unidirectional secretion of digestive enzymes and fluid into the pancreatic duct.

In the case of epithelial cells, the key molecular mechanisms that establish polarity are understood in terms of polarity determinant complexes that include a tight junctional complex known as the PAR-3–PAR-6–aPKC (atypical protein kinase C) complex (Goldstein and Macara, 2007) and a basal complex of Dlg­–Lgl–scribble (Humbert et al., 2003). However, there are many cell types, such as endocrine cells, where functions are located in distinct cellular regions (Moser and Neher, 1997), but where it is unknown if these specialisations are located by mechanisms of polarity. A good example is the insulin-secreting pancreatic β-cell. It is known that the main glucose uptake transporter, GLUT2 (also known as SLC2A2), is located on the lateral membrane between the cells (Orci et al., 1989). Evidence also indicates that insulin secretion selectively occurs at the vascular face of the β-cells (Low et al., 2014). These segregated functions imply that β-cells have polarity, and work on polarity pathways, such as the LKB1–AMPK pathway (Fu et al., 2009; Granot et al., 2009; Kone et al., 2014), or viral budding (Lombardi et al., 1985) support this idea. However, islets of Langerhans are a compact mass of thousands of cells and do not have obvious physical boundaries and domains, such as lumens that are found in epithelial tissues. As such, it is unclear whether β-cells have a consistent orientation in the islet and it is unknown whether they possess the classical polarity determinants that might underpin regional specialisations.

Here, we have used a pancreatic slice preparation that maintains the native structural organisation of the islets (Marciniak et al., 2014). We have imaged in three dimensions, using three distinct methods; live-cell two-photon microscopy, immunofluorescence confocal microscopy and, finally, serial block-face electron microscopy. Together, these methods provide new insights into the *in situ* organisation of β-cells, and show that they are consistently orientated with respect to the vasculature with polarity determinants that define three distinct domains.

## RESULTS

### β-cells possess at least two distinct functional domains

**Fig. 1.**
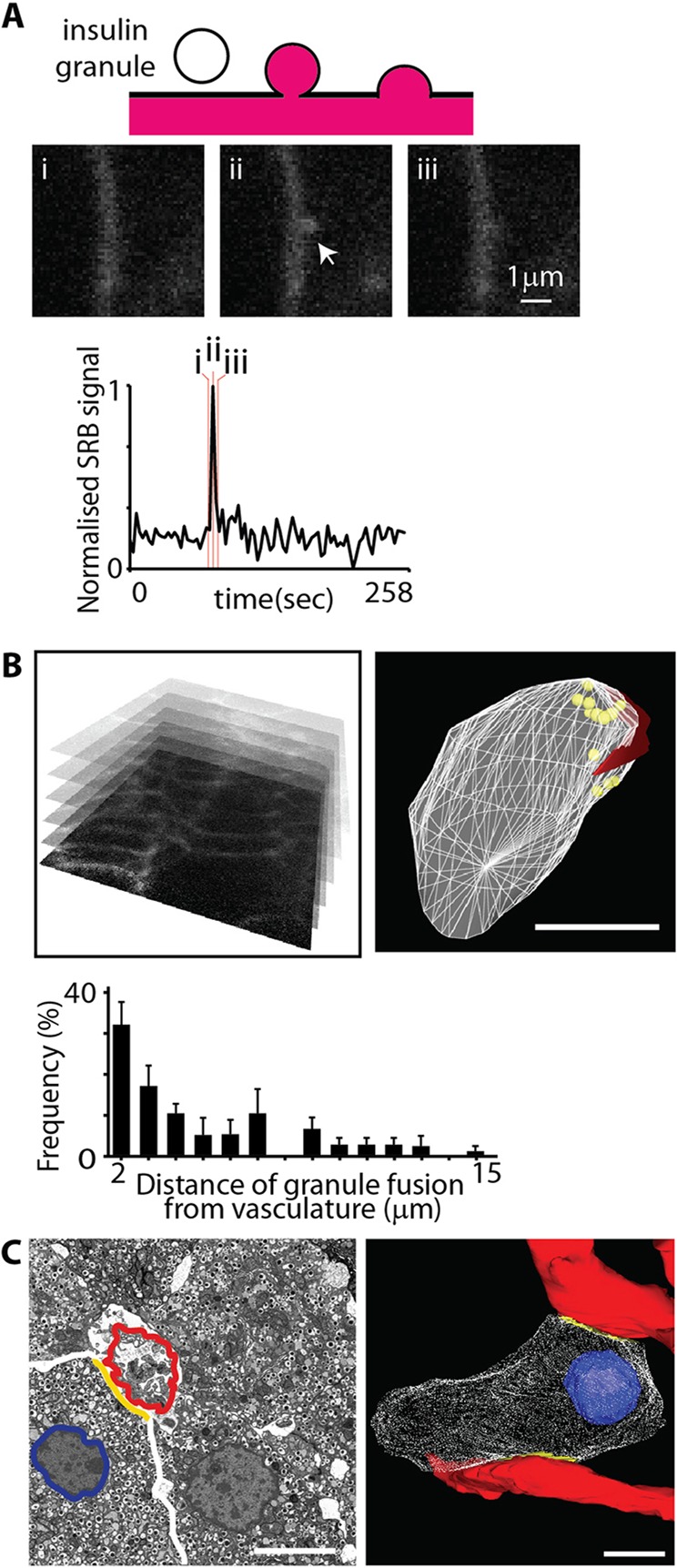
**β-cells are arranged with a consistent orientation with respect to the islet blood vessels.** (A) Live-cell 3D two-photon microscopy tracks the rapid influx of sulforhodamine B (SRB) dye into fusing granules, previously characterised as insulin-containing granules, in response to a 15 mM glucose stimulus (Low et al., 2013). An individual fusion event is shown at three times (i, ii, iii) with the fluorescence intensity over time, within a region of interest centred over the granule, showing a sharp peak and rapid decay. (B) Data collected in 3D, with 2 µm between each section, showing the location of each granule fusion event as yellow circles. The basement membrane marker isolectin B4 (shown in red) identifies the region of the β-cell adjoining the vasculature. 3D analysis of the distribution of granule fusion distances shows a strong bias towards the vasculature. (C) Serial block-face scanning electron microscopy (single image from the stack shown on the left) identified the 3D relationships between the blood vessels (red) and β-cells, with the contact points (yellow) defining a small membrane domain (3D reconstruction shown on the right). The is a total of 19 cells in this volume, and of these eight cells make single contact with vasculature and 11 cells make two points of contact. Scale bars: 5 µm (B,C).

Most of the islet volume consists of endocrine cells packed in close contact with each other, but most endocrine cells also make contact with the capillary blood vessels (Weir and Bonner-weir, 1990). The contact points of the β-cells to the vasculature have been proposed, based on the distribution of insulin granules, to be the site of insulin granule exocytosis (Bonner-Weir, 1988). We provide direct functional evidence for this, by using three-dimensional (3D) live-cell imaging of glucose-induced insulin granule exocytosis and by employing a two-photon granule fusion assay (Fig. 1A; Low et al., 2013, 2014). Insulin granule fusion, in response to glucose stimulation, was strongly biased towards the blood vessels, in this case stained with isolectin B4 (Fig. 1B). To further characterise these β-cell–vascular contact points we used 3D electron microscopy, employing serial block-face sectioning of intact fixed mouse islets (Fig. 1B). All cells made one point of contact and many (11 out of 19 cells in this block) had two points of contact with the vasculature. The total area of this contact was proportionately small compared to the total cell membrane area of the β-cells (vascular contact area is 7.9±3.8%, mean±s.e.m., *n*=19 cells, Fig. 1C).

**Fig. 2.**
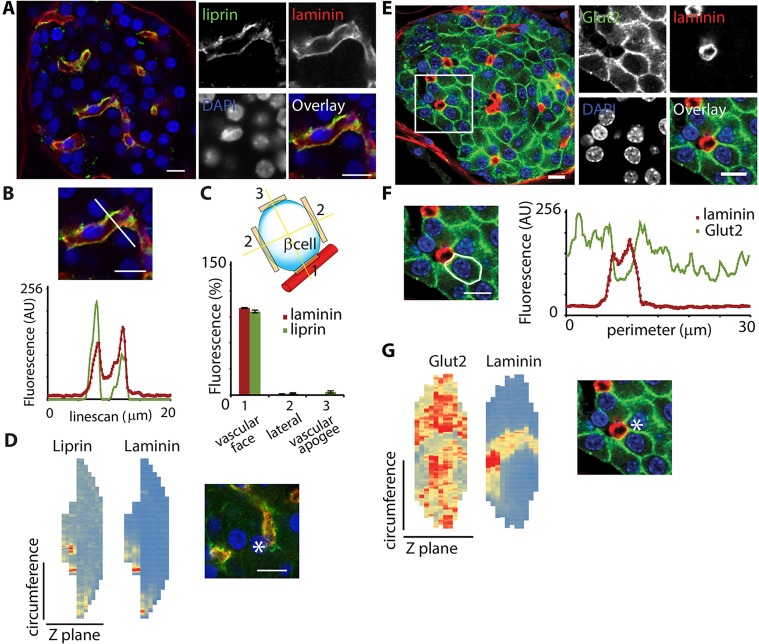
**Enrichment of the synaptic protein liprin and GLUT2 in specific and distinct domains.** (A) Immunofluorescence shows that the synaptic scaffold protein liprin (green) is enriched along the vascular face of β-cells (labelled with laminin, red). (B) A linescan drawn across a blood vessel shows the coincidence of liprin and laminin location; previous work has shown that liprin is expressed in β-cells (Low et al., 2014). AU, arbitrary units. (C) A schematic of a β-cell illustrates a division of the cell membrane into three domains. Linescan analysis of the peak fluorescence in each domain quantitatively identifies enrichment of liprin at the vascular domain (mean±s.e.m.; *n*=14 cells within one islet, representative of the distribution in islets from six animals). (D) Heatmap representation (blue is low fluorescence, red is high fluorescence) of liprin and laminin distribution, using fluorescence intensities along cell circumference. Linescans at each *z*-stack show coincident enrichment of both proteins at the vascular face of the β-cell. (E,F) Immunofluorescence of GLUT2 (green) shows enrichment along the lateral regions away from the vasculature, immunostained for laminin (red). (G) A heatmap representation, using cell circumference linescan analysis at each *z*-stack, shows that GLUT2 is widespread across the cell surface, but laminin has a relatively discrete enrichment. The asterisk in D and G indicates the cell used in the heatmap analysis. Scale bar: 10 µm.

As well as being the domain of targeted exocytosis, the regions of β-cells that contact the vasculature were also enriched in synaptic scaffold proteins, like liprin α1 (Fig. 2A–D; Movie 1), as well as other synaptic proteins such as RIM2 (also known as RIMS2), ELKS (also known as ERC1) and piccolo (Low et al., 2014). Thus, both functional and structural evidence indicate this region is a distinct β-cell domain specialised for secretion. This domain at the vascular face is separate from the distribution of GLUT2, which is on the lateral regions between cells (Fig. 2E–G; Movie 2). GLUT2 is the main glucose influx pathway in rodents and the segregation of this domain from the secretory domain suggests a hitherto unrecognised importance to the spatial organisation of the stimulus secretion pathway.

### β-cells show a consistent orientation with respect to the vasculature

The above data suggest that there is a 3D organisation of β-cells and a consistent spatial relationship with the vasculature of the islet. As an aid to understanding these relationships, we further analysed our immunofluorescence data from islet slices. First, in single planes, we defined three separate plasma membrane domains: (1) the vascular face, identified by the colocalisation with the basement membrane protein laminin; (2) the lateral domains along the sides of the cells; and (3) the vascular apogee, identified as the region of cell membrane furthest away from the vasculature (Fig. 2C). A linescan analysis across each of these membrane domains, as shown in Fig. 2B, identified the peak fluorescence, which, when plotted out, showed an enrichment of liprin at the vascular face, as identified with laminin (Fig. 2C). Second, we made a 3D reconstruction of the cell surface distribution of liprin and laminin using linescans around the cell circumference at each image *z*-plane, with fluorescence intensity represented as a heatmap (Fig. 2D). Both approaches showed that liprin was specifically enriched along the vascular face of the β-cells and demonstrate a consistent orientation of β-cells with respect to the vasculature, suggesting that β-cells are polarised.

We performed a similar analysis for the 3D distribution of GLUT2, and again used laminin as a marker for the vascular face (Fig. 2E–G). GLUT2 was specifically enriched along the regions away from the vasculature where there are endocrine–endocrine contacts, as shown in the 2D linescans (Fig. 2F) and the 3D cell circumference heatmap (Fig. 2G).

### Evidence for a third spatial domain in β-cells

**Fig. 3.**
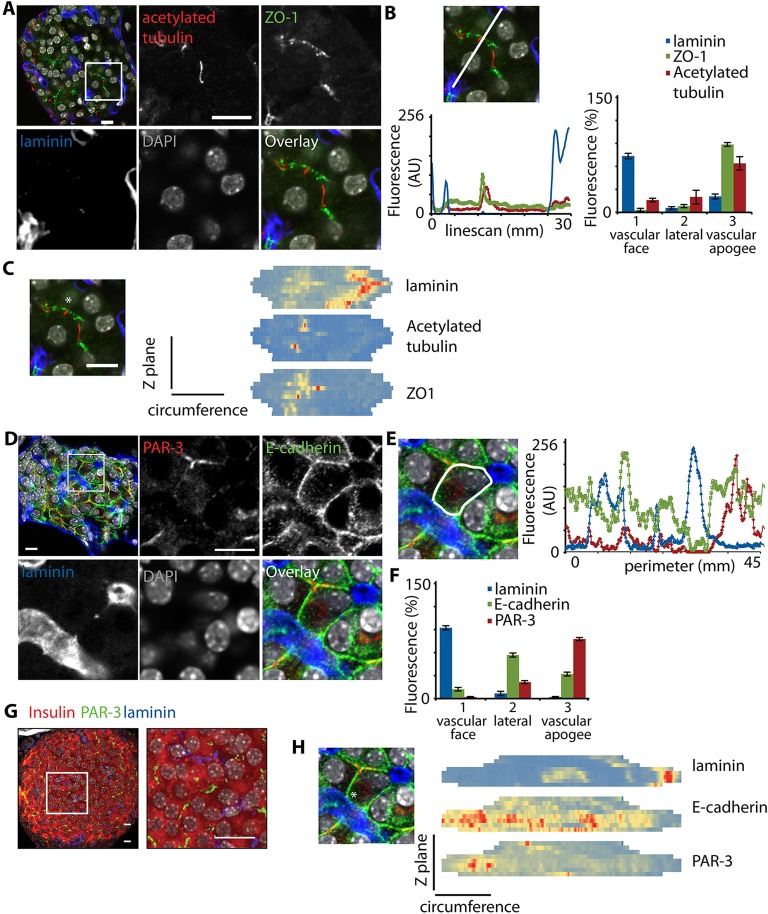
**Identification and characterisation of an apical domain in β-cells.** (A) Immunostaining of acetylated tubulin (as a marker for primary cilia, red) and ZO-1 (green) shows enrichment at the pole of the β-cell that lies away from the vasculature (labelled with laminin, blue). (B) Linescan and distribution analysis demonstrates the enrichment of acetylated tubulin and ZO-1 in domain 3, at the vascular apogee (mean±s.e.m.; data from 13 cells from two islets, representative of ZO-1 in islets from six animals, and tubulin in islets from eight animals). (C) A heatmap representation, using cell circumference linescan analysis at each *z*-stack, shows that ZO-1 and acetylated tubulin have a coincident enrichment at the opposite side of the cell to laminin. (D) The apical determinant PAR-3 (red) is enriched at the pole of β-cells that lies away from the vasculature (labelled with laminin, blue) and contrasts with the distribution of E-cadherin (green), which is found along the lateral membrane domains. (E) A linescan drawn around the perimeter of a single cell (white line) shows the relative distribution of these proteins. (F) This is further quantified with a linescan analysis of immunofluorescence across the membrane domains of the β-cells, which shows E-cadherin enriched on the lateral membrane and PAR-3 at the vascular apogee (mean±s.e.m.; *n*=13 cells from one islet, representative of PAR-3 in islets from eight animals, and E-cadherin in islets from four animals). (G) Immunolocalisation of PAR-3 (green) with insulin (red) in whole islets (left). The right panel shows a magnified image. (H) A heatmap representation, using cell circumference linescan analysis at each *z*-stack, showing the widespread distribution of E-cadherin and the enrichment of PAR3 that is positioned away from the enrichment of laminin. In C and H, the asterisk identifies the cell used in the heatmap analysis. Scale bars: 10 µm.

Recent work has highlighted the importance of primary cilia in β-cell function (Gerdes et al., 2014). Primary cilia are often located in the apical region within a spatial domain defined by tight junctions. We therefore used our methods, in pancreatic slices, to determine whether primary cilia and tight junctions in β-cells also have a consistent orientation in the islet. We found that the tight junction protein zona occludens 1 (ZO-1, also known as TJP1) and acetylated tubulin, a marker for primary cilia, were present in cells in the islet (Fig. 3A) with both proteins positioned at the vascular apogee (Fig. 3B,C; Movie 3) indicting this region as a likely apical domain. Re-analysis of the electron microscopy data in Fig. 1C confirmed that the primary cilia was located away from the two blood vessels (Fig. S3A). In total therefore, we suggest that the β-cells possess three functionally distinct domains; apical, lateral and basal. Our 3D analysis indicates these domains are consistently orientated with respect to the vasculature and imply an underlying cellular polarity.

### Apical polarity determinants define an apical region in the β-cell that is opposite to the vascular face

If β-cells really are polarised then they might be expected to possess the determinants of polarity that are found in epithelial cells. Using islet slices and immunostaining (Low et al., 2014; Meneghel-Rozzo et al., 2004), we determined whether the islet cells possess the classical apical determinant PAR-3 (also known as PARD3) (Fig. 3D). The images show that PAR-3 was consistently located away from the laminin-stained vasculature and is relatively discrete, occupying a small domain of the cells; this contrasts with E-cadherin staining, which is enriched along the entire lateral membrane (Fig. 3D; Movie 4). Using linescan analysis and domain distribution, as well as 3D circumference heatmaps, to quantify the protein locations it was clear that both were excluded from the β-cell vascular face, that E-cadherin is enriched on the lateral domain, and that PAR-3 is enriched at the vascular apogee which, given this defining feature, we will now term the apical domain (Fig. 3E,F,H). Fig. 3G shows that PAR-3 was present in β-cells, which, in these experiments were counter-immunostained with insulin.

**Fig. 4.**
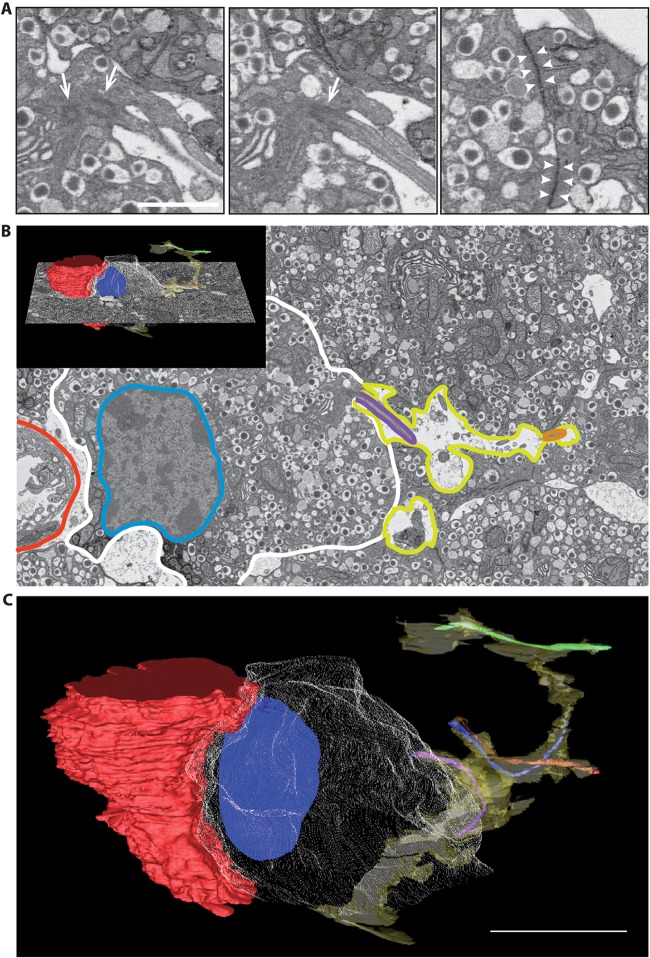
**Serial electron microscopy defines an apical domain and supracellular luminal volume.** (A) Enlarged regions from single electron microscopy sections showing the centrioles (arrows) at the base of an example primary cilia and the putative tight junctions (arrowheads). (B) 50-nm serial sections were then used to construct a model in IMOD that highlights a single β-cell (mesh outline, blue nucleus) within an islet. This cell contacts the blood capillary (red) on the left. Regions of close apposition between adjacent β-cells, consistent with tight junctions were used to outline a contiguous extracellular space (yellow outline) suggestive of a discrete luminal compartment in which adjacent β-cells also place their primary cilia (green, blue, orange and purple). (C) The full-depth model of the single cell shows its relationship to the vasculature and the luminal space. Blood capillary, red; β-cell, white; nucleus, blue; extracellular luminal space, yellow; primary cilia, green, blue, orange and purple lines. Scale bars: 1 μm (A), 5 μm (C).

To increase our spatial resolution of this apical domain, we turned again to serial block-face electron microscopy. Using 50-nm thick sections, through a depth of 25 µm, we were to identify the orientation and components of the putative apical domain. The region of the vascular apogee shows evidence for contact points of close apposition between β-cells that are consistent with tight junctional links (Fig. 4A, arrowheads) and were used to provide the outline volume of an extracellular apical lumen (yellow, Fig. 4B,C). Projecting into this lumen are primary cilia that show evidence for centrioles at their bases (Fig. 4A, arrows). Each serial section (Fig. 4B; Fig. S1) was then used to produce a reconstructed image, drawn from all the sections within the volume, which shows the vasculature (red) on the left, and a single exemplar cell outlined in a mesh (grey) with its nucleus (blue) and cilia from four adjacent β-cells (green, orange, blue and purple) that all project into the extracellular luminal space (Fig. 4C; Movie 5). Together, our data indicate that tight junctions and primary cilia define a discrete spatial domain in β-cells that lies opposite to the vasculature.

### Basolateral polarity determinants define the lateral regions between β-cells and the vascular face

**Fig. 5.**
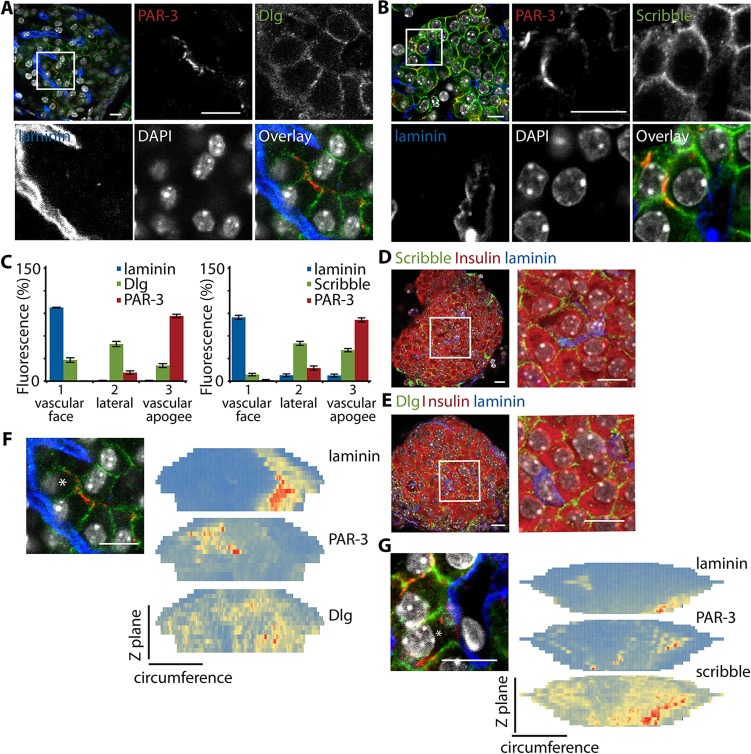
**Identification of the basal region of β-cells using Dlg and scribble.** (A,B) Immunostaining with PAR-3 and Dlg or scribble shows a predominantly lateral location of Dlg and scribble. (C) This was quantitatively confirmed, using linescan analysis, and identifies relative enrichment of both Dlg and scribble in the lateral domain. Counter-immunostaining for PAR-3 showed the characteristic enrichment in the vascular apogee, as before (mean±s.e.m.; Dlg, *n*=20 cells from three islets; scribble, *n*=15 cells from two islets; results are representative of Dlg in islets from four animals, and scribble in islets from seven animals). (D,E) Co-immunolocalisation of scribble or Dlg (both green) with insulin (red) in whole islets (left). Magnified images are shown on the right. (F,G) Heatmap representations, using cell circumference linescan analysis at each *z*-stack, showing that the discrete enrichment of laminin (identifying the vascular face) is separated from the enrichment of PAR-3 (apical region), and that both Dlg and scribble have a relatively widespread location across the cell. Scale bars: 10 µm.

Given that the above experiments define an apical region away from the vascular face, we next set out to determine the presence and location of protein determinants of the basal domain. Immunostaining for either Dlg family proteins (Fig. 5A) or scribble (Fig. 5B) showed that these were located around the β-cell membrane, with a particular enrichment along the lateral surfaces (Fig. 5C,F,G; Movies 6 and 7). We found, using counter-immunostaining, that these basal polarity determinants were located in insulin-positive β-cells (Fig. 5D,E), and conclude that these basal determinants provide further evidence that β-cells are systematically orientated with respect to the vasculature and can be considered as polarised cells.

This polar organisation of β-cells within islets was conserved in humans. Immunostaining of human islets showed that PAR-3 was located in the vascular apogee of insulin-containing β-cells, consistent with the mouse data (Fig. S2A). We also found that scribble was located in the lateral and basal regions of insulin-containing β-cells (Fig. S2C). Quantitative assessment of the distribution of these proteins (Fig. S2B) shows a similar distribution to that of β-cells in the mouse islets.

## DISCUSSION

Here, we show that pancreatic β-cells maintain a consistent orientation with respect to the islet capillaries that is defined by apical and basal regions and the positioning of polarity determinants, like PAR-3 and scribble. Our approach employs a pancreatic slice method (Marciniak et al., 2014) that preserves the native islet organisation, unlike the more widely used islet cultures which can cause structural changes [e.g. an increase in tight junctions induced by the enzyme treatments (Intveld et al., 1984)] and loss of endothelial cells (Lukinius et al., 1995). Our pancreatic slice method is rapid and uses enzyme inhibitors, and is therefore likely to closely reflect the native organisation and expression of tight junctional proteins.

Subsequent imaging with either confocal or block-face serial electron microscopy has enabled us to build up a comprehensive picture of the 3D arrangement of β-cells within the islet. We quantify the 3D spatial distribution of polarity determinants to demonstrate three distinct domains in β-cells. First, an apical region, identified by enrichment of PAR-3 and ZO-1, which encompasses an extracellular ‘lumen’ into which project primary cilia. Second, a lateral region enriched with scribble and Dlg that co-localises with the GLUT2 transporter. And, finally, a basal region where β-cells contact the vasculature and show enrichment of synaptic scaffold proteins like liprin. The compartmentalised location of these structural and functional proteins suggests that polarity regulates β-cell function.

### β-cell polarity defines three distinct domains

#### The distinct apical domain in β-cells

Our data show a supra-cellular organisation that links tight junctions from one cell to another, which together, circumscribe an extracellular volume into which primary cilia project from a number of adjoining β-cells (Fig. 4). Given the sensory function of primary cilia (Singla and Reiter, 2006), and recent work suggesting that they are the site of enrichment of insulin receptors (Gerdes et al., 2014), this definition of a new domain within the islets has widespread implications for autocrine and paracrine signalling.

#### The lateral domain

Our data show a consistent local enrichment of scribble and Dlg along the lateral membrane that lies between β-cells and separates the apical region at the vascular apogee from the basal region where the β-cells contact the vasculature. This is also the region of enrichment of E-cadherin and GLUT-2. The significance of separating a region of glucose uptake, away from the vasculature, where insulin secretion occurs (Low et al., 2014), adds a new level of insight into the stimulus–secretion coupling cascade in β-cells and is likely to be functionally significant in the control of insulin secretion.

#### The distinct basal region that contacts the vasculature

We suggest that the most significant interaction is between the β-cells and the basement membrane that is secreted by the endothelial cells. Our imaging shows that the basolateral determinants Dlg and scribble are present in this region, although, as in epithelial cells, they are less abundant than along the lateral domain. Past work has shown that this is the region where the majority of insulin granule exocytosis occurs, which would therefore target insulin delivery into the blood stream (Low et al., 2014). The spatial segregation of this ‘secretory’ region from the lateral and apical domains, once again, provides significant new insights into β-cell function and islet structure. For example, targeting insulin secretion into the vasculature spatially segregates insulin detection at the cilia, which would minimise autocrine communication and make the β-cells responsive to circulating insulin, as predicted in a recent modelling paper (Wang et al., 2013).

### Comparison with past work – apical and lateral regions

We suggest that the luminal extracellular space we identify here is the same as the previously identified canaliculi (Yamamoto and Kataoka, 1984). The original description, using electron microscopy, showed that canaliculi contain microvilli and cilia and are bordered by tight junctions (Yamamoto and Kataoka, 1984). The colocalisation of ZO-1, acetylated tubulin and PAR-3 that we now show is good evidence that this apical domain is defining the same extracellular space as the previously described canaliculi. This has interesting implications for β-cell function given that we are now making a spatial distinction between this apical domain, which forms a discrete region, and the much larger lateral surface.

In our model, both the apical domain and lateral domain must have functional continuity with the blood. For the apical region, this is needed for sensing of insulin, and maybe other factors, by the cilia, and in the lateral region it is needed for the uptake of glucose by GLUT2. We suggest that the tight junctions, which are known to be labile (Intveld et al., 1984), might be modulated by different physiological inputs, such as glucose (Orci, 1976) and therefore could be functionally important in selectively restricting diffusional access to the apical lumen.

Our immunostaining of GLUT2 is consistent with a previous report showing that it occupies the large lateral surfaces (Thorens et al., 1990). However, other work suggests the GLUT2 transporter is enriched on microvilli (Orci et al., 1989), which, in the context of our model, would place it in the apical domain. However, the work of Orci et al. shows that GLUT2 is also present on the flat membrane lying between the cells, a region we would classify as lateral. Given that this lateral region is much more extensive than the apical region, it could be functionally dominant for the sensing of glucose. Further work is needed to clarify this point because the specific site of enrichment of functionally important GLUT2 transporters in the apical or lateral domains has major implications for the control of β-cell behaviour.

Recent work on cultured islets suggests that there is F-actin enrichment along cell ‘edges’ between β-cells (Geron et al., 2015) that are also enriched in proteins, such as GLUT2 or SNAP25. Our data here, and elsewhere (Low et al., 2014) argues that these proteins are present relatively uniformly across the entire lateral surface of cell-to-cell contacts. The differences might be due to the use of cultured islets versus acute slices; if so, the study of any structural reorganisation could give useful insights into the mechanisms that are needed to build β-cell architecture.

### Comparison with past work – basal region

β-cell contacts with the endothelial basement membrane are the site of laminin–integrin interactions, which are important for β-cell proliferation (Nikolova et al., 2006). Here, we show that this same region contains Dlg and scribble, classical markers for basal domains, and is the site for preferential fusion of insulin granules. Whether insulin secretion can be spatially targeted has been controversial with evidence for (Bokvist et al., 1995; Bonner-Weir, 1988; Paras et al., 2000) and against (Rutter et al., 2006; Takahashi et al., 2002) targeting. Our live-cell 3D imaging, which we performed *in situ* within intact islets, now precisely maps the β-cell to vasculature contacts and provides direct evidence for a strong bias of exocytosis at the vascular face (Fig. 1; Low et al., 2014).

The arrangement of β-cells around a capillary has been described as a rosette (Bonner-Weir, 1988). Such rosettes are apparent in some of our images but the 3D complexity of the islet blood vessels means that in many cross-sections this organisation is not clear. The seminal paper of Bonner-Weir (1988) suggested that the majority of β-cells make two points of contact with blood vessels, which is consistent with our analysis. It is attractive to speculate that this represents one point of arteriole contact and one point of veniole contact but there is little evidence to support this idea.

### Comparison with past work – polarity

Past work has discussed β-cell polarity in terms of nuclear position and location of cilia (Granot et al., 2009; Kone et al., 2014; Sun et al., 2010). The position of the primary cilia, taken as a proxy for the apical domain, led to the suggestion that the β-cells are more similar to hepatocytes (Granot et al., 2009), with apical regions situated along the lateral surfaces, than to a classical columnar epithelial organisation, where the apical domain is opposite to the basal domain.

Our 3D analysis now extends our understanding and shows further complexity to β-cell polarity. We show that, in terms of area, the largest domain of β-cells is the lateral surface formed at endocrine–endocrine cell contacts. Within this lateral surface is a discrete apical domain. The contiguous alignment of apical domains from adjacent cells forms an extracellular lumen. This apical region is positioned at the furthest distance away from the points of vascular contact(s). These sites of vascular contact, we propose, form a distinct basal surface; in this way two points of contact would lead to two basal surfaces. This suggests that β-cells can have multiple basal and apical surfaces embedded within the larger area of the lateral surface. Cartoons representing our proposed cell orientation with respect to the vasculature are shown in Fig. S3B. Work in other systems is expanding our understanding of cell polarity to include cell types with multiple apical domains (Denker et al., 2013), and perhaps pancreatic β-cells represent another extension of this diversity. Finally, our work indicates that the polar organisation of β-cells we find in the mouse islet is recapitulated with a similar organisation in the human despite the fact that other significant differences exist between mouse and human islets (Cabrera et al., 2006).

### Conclusions

We conclude that β-cells are structurally and functional subdivided into three distinct domains. This cell polarisation spatially separates cell functions, and our work provides a framework for future work into understanding β-cell control. The next stage in exploring β-cell polarity will require further technical advances that enable routine manipulation of β-cells *in situ* and potentially the use of *in vitro* morphogenetic 3D models, such as are used in epithelial cell biology (Cerruti et al., 2013).

## MATERIALS AND METHODS

### Experimental solution

Experiments were performed in Na-rich extracellular solution (in mM: 140 NaCl, 5 KCl, 1 MgCl_2_, 2.5 CaCl_2_, 5 NaHCO_3_, 5 HEPES, 3 mM glucose) adjusted to pH 7.4 with NaOH.

### Islet preparation

Mice were humanely killed according to local animal ethics procedures (approved by the University of Queensland, Anatomical Biosciences Ethics Committee). Human islet slices were obtained from the Network for Pancreatic Organ Donors with Diabetes (nPOD) tissue bank that contains cryopreserved tissue sections that had been fixed with paraformaldehyde and then immunostained following the protocol below.

### Islet slices

Sectioning of unfixed pancreatic tissue was performed as described by Huang et al. (2011). Briefly, after cervical dislocation, the pancreas of 10–12-week-old CD1 male mice was injected with 1.9% low-melting-point agarose (UltraPure LMP, Invitrogen) in extracellular slice medium (ECSM, 125 mM NaCl, 2.5 mM KCl, 1.25 mM NaH_2_PO_4_, 26 mM NaHCO_3_, 2 mM sodium pyruvate, 0.25 mM ascorbic acid, 2 mM myo-inositol, 1 mM MgCl_2_, 2 mM CaCl_2_, 6 mM lactic acid and 6 mM glucose at pH 7.4). The common bile duct was clamped at the junction with the duodenum to prevent agarose from entering the small intestine and a 30-gauge needle was used to inject 1–3 ml of 42°C agarose through the bile duct to backfill the pancreas. The pancreas was immediately cooled with ice-cold ECSM, removed from the mouse, and immersed in ice-cold ECSM in a petri dish. 4- to 6-mm cubes of this tissue were embedded in 4% low-melting-point agarose in ECSM, immersed in 4°C ECSM and sectioned with a Zeiss (Thermo-Fisher) Hyrax V50 vibrating microtome. Sections (90–100-μm thick) were cut with the instrument set at an amplitude of 0.7, frequency of 95 and a speed of 4 μm/s, and sections containing uncut islets were stored in ECSM [oxygenated by bubbling with 5% carbogen gas and supplemented with 0.1 mg/ml trypsin inhibitor (Sigma)] at 4°C for no longer than 10 min before fixation. Fixation with 4% paraformaldehyde (Sigma-Aldrich) in ECSM was either for 10 min (short PFA) or 1 h (long PFA) at 20°C. Slices fixed with methanol were rapidly immersed in −20°C methanol and stored in a freezer for 1 h. Methanol-fixed slices were rehydrated in ECSM and then PBS. Slices were stored in either PBS or ECSM at 4°C for up to 1 week before antibody treatment.

Immunofluorescence was performed as described by Meneghel-Rozzo et al. (2004). Sections were incubated in blocking buffer (3% BSA, 0.3% donkey serum, 0.3% Triton X-100) for a minimum of 1 h at room temperature followed by primary antibody incubation (see below) at 4°C overnight in blocking buffer. Typically four to six slices were incubated in 0.5 ml blocking buffer in one well of a six-well dish. Sections were washed in PBS (four changes over 30 min) and secondary antibodies (in blocking buffer) were added for 4–6 h at 20°C. After washing in PBS, sections were mounted in Prolong Gold anti-fade reagent (Invitrogen) and imaged on an Olympus Fluoview FV1000 confocal microscope using a UPlanSApo 60×1.35 NA oil objective.

The linescan analysis, for example in Fig. 2B, identified the peak fluorescence at, or close to, the membrane, which was then averaged to produce the distribution plots shown, for example, in Fig. 2C. The heatmap representations (for example in Fig. 2D) used fluorescence intensities along linescans around the cell circumference at each *z-*section. The resultant array of fluorescence intensities was then normalised to the brightest region and shown on a blue–yellow–red (fluorescence intensity 0–100%) colour scale.

### Serial block-face electron microscopy

Pancreatic slices were fixed in 2.5% glutaraldehyde, washed in PBS and double post-fixed by using 2% OsO_4_ with 1.5% potassium ferricyanide followed by 1% thiocarbohydrazide and then another 2% OsO_4_. Samples were stained overnight with 1% uranyl acetate and then for 1 h at 60°C in Walton's lead aspartate. They were then serially dehydrated with acetone and embedded with Durcapan resin and polymerised. Individual islets were cut out of the resin, mounted and then imaged and sectioned using a Zeiss Sigma SEM fitted with a 3View (Gatan, CA, USA) at 2.25 kv and 10 Pa. The resultant images were analysed using the programme IMOD (Kremer et al., 1996) and 3D reconstructions performed.

### Antibodies

Primary antibodies used for this study were: rat anti-β1 laminin (MAS5-14657, Thermo Scientific), mouse anti-Dlg (610874, BD Transduction Laboratory), mouse anti-E-cadherin (610181, BD Transduction Laboratory), mouse anti-insulin (I2018, Sigma), rabbit anti-PAR-3 (07-330, Merck Millipore), goat anti-Scrib (sc-11049, Santa Cruz Biotechnology) and rabbit anti-PPFIA1 (liprin 1α 14175-1-AP Proteintech). All primary antibodies, except the laminin antibody, were diluted 1:200, the anti-laminin antibody was used at 1:500. Secondary antibodies were highly cross-absorbed donkey or goat antibodies (Invitrogen) labelled with Alexa Fluor 488, Alexa Fluor 546 or Alexa Fluor 633. All were used at a 1:200 dilution. Where used, DAPI (Sigma, 100 ng/ml final concentration) and Alexa-Fluor-633–phalloidin (A22284, 2 U/ml final concentration, Invitrogen) were added for the last 2 h of secondary antibody incubation.

### Statistical analyses

All numerical data are presented as mean±s.e.m. Statistical analysis was performed using Microsoft Excel and GraphPad Prism. Data sets with two groups were subjected to a two-tailed, un-paired Student's *t*-test. Islets from at least 3 animals were used in each experiment.
